# Novel dihydroartemisinin derivative DHA-37 induces autophagic cell death through upregulation of HMGB1 in A549 cells

**DOI:** 10.1038/s41419-018-1006-y

**Published:** 2018-10-15

**Authors:** Xiufeng Liu, Juanjuan Wu, Menglin Fan, Chen Shen, Wenling Dai, Yini Bao, Ji-Hua Liu, Bo-Yang Yu

**Affiliations:** 1 State Key Laboratory of Natural Medicines, School of Traditional Chinese Pharmacy, China Pharmaceutical University, 210009 Nanjing, China; 2 Jiangsu Key Laboratory of TCM Evaluation and Translational Research, China Pharmaceutical University, 210009 Nanjing, China

## Abstract

Dihydroartemisinin (DHA) and its analogs are reported to possess selective anticancer activity. Here, we reported a novel DHA derivative DHA-37 that exhibited more potent anticancer activity on the cells tested. Distinct from DHA-induced apoptosis, DHA-37 triggered excessive autophagic cell death, and became the main contributor to DHA-37-induced A549 cell death. Incubation of the cells with DHA-37 but not DHA produced increased dots distribution of GFP-LC3 and expression ratio of LC3-II/LC3-I, and enhanced the formation of autophagic vacuoles as revealed by TEM. Treatment with the autophagy inhibitor 3-MA, LY294002, or chloroquine could reverse DHA-37-induced cell death. In addition, DHA-37-induced cell death was associated significantly with the increased expression of HMGB1, and knockdown of HMGB1 could reverse DHA-37-induced cell death. More importantly, the elevated HMGB1 expression induced autophagy through the activation of the MAPK signal but not PI3K-AKT–mTOR pathway. In addition, DHA-37 also showed a wonderful performance in A549 xenograft mice model. These findings suggest that HMGB1 as a target candidate for apoptosis-resistant cancer treatment and artemisinin-based drugs could be used in inducing autophagic cell death.

## Introduction

Non-small-cell lung cancer (NSCLC) accounts for 85–90% of lung cancer deaths due to relatively insensitive or development of resistance to chemotherapy^1,2^. Many attempts have been made to develop novel chemotherapies either by exploring the anticancer ability of novel compounds or by assessing drugs conventionally used in other clinical diseases. Traditional Chinese medicine (TCM) have been known to be effective against a range of diseases and considered to be a natural source of novel and potent anticancer drugs with minimal side effects in clinical. Artemisinin (ART), as one of the promising compounds, which is isolated from traditional Chinese herb and has been used for more than 2000 years, has profound effects on malaria and parasitic diseases^3,4^. It has been found that artemisinin and its derives also have potent anticancer activity^5,6^. Among these derives, artesunate and DHA are considered to be the most active compounds and subsequently many researchers have been focused on developing novel compounds with enhanced activity, increased selectivity, and low toxicity in vitro. In our previous study, a series of DHA derives were synthesized by the combination of biotransformation and chemical modification. Among them, DHA-37 exhibited an excellent anticancer activity compared with DHA or other derivatives^7,8^. However, the molecular mechanism of DHA-37-induced cell death needs to be further studied.

For a long time, promoting apoptosis has been used as a main strategy for cancer drug discovery. However, many tumors are not sensitive to drug-induced apoptosis, and also the acquisition of resistance to therapy is becoming an important clinical problem^9,10^. It is not always possible to work, although many strategies were conducted to overcome the apoptosis resistance, such as, increasing the expression of anti-apoptotic proteins, downregulation, or mutation of pro-apoptotic proteins^11^. Accumulating evidence has shown that inducing autophagic cell death may be a promising therapeutic approach and might offer a new hope for treating apoptosis resistance tumor^12,13^. Autophagy has paradoxical roles in adjusting both cell death and survival during tumor development and cancer therapy. It has been reported that excessive autophagy can cause cell death and several agents were reported to induce autophagic cell death in different cancer cell types^14–16^. Inducing autophagic cell death is becoming an attractive approach for anticancer therapies.

High mobility group box 1 (HMGB1) could translocate from nucleus to cytoplasm to play as damage-associated molecular pattern molecules (DAMPs) and modulate various physiological and pathological processes^17–19^. Recently, the role of HMGB1 in autophagy has been studied by different research groups. The result from Tang et al. revealed that autophagy is dependent on HMGB1^20,21^. When the cells are treated by starvation or stimulated by autophagy inducer, HMBG1 could interact with Beclin1 to dissociate it from BCL2 and then cause autophagy^22^. This conclusion was also provided in the HMGB1 conditional knockout mouse models^23^. However, the conditional liver knockout study from Schwabe’s group showed that HMGB1 is independent for autophagy^24,25^. So, further studies are needed to clarify the relationship between HMGB1 and autophagy, especially in different cell or tissue types. Overall, although the role of HMGB1 in autophagy is complex and the exact mechanism is not clear, HMGB1 is becoming an attractive target for anticancer therapies.

In the present study, the sensitivities of different human cancer cells to DHA and its derivatives DHA-37 were compared. The mechanism study revealed that inducing autophagic cell death but not apoptosis or programmed necrosis is the main reason for DHA-37-induced cell death. Further, the relationships between DHA-37-induced HMGB1 upregulation and autophagic cell death were investigated in A549 non-small-cell lung carcinoma cells and the signaling pathways involved in DHA-37-induced autophagic cell death were investigated. Finally, the anticancer activity of DHA-37 was validated in vivo in a human A549 lung cancer xenograft model. Our findings may provide novel insights into the mechanisms underlying the anticancer effects of the artemisinin and its analogs against non-small-cell lung carcinoma cells.

## Results

### DHA-37 is significantly more potent than DHA in killing various human cancer cells

**Fig. 1:**
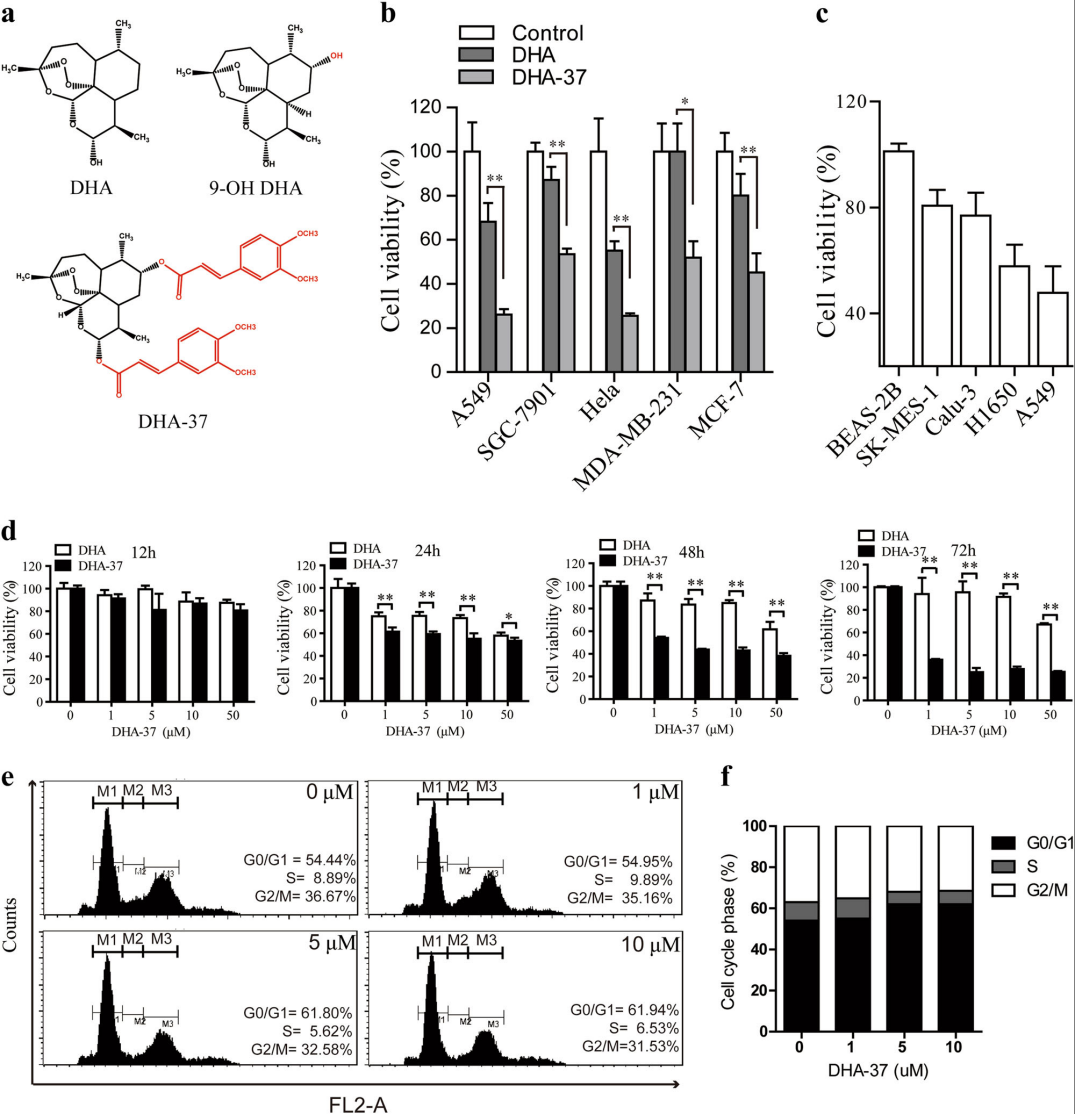
DHA-37 induces cell death in a more efficient way than DHA does. **a** Chemical structure of DHA (dihydroartemisinin), 9-OH DHA, and DHA-37. **b** Diferent human cancer cell lines were treated with 10 μM DHA or DHA-37 for 48 h. Cell viability was assessed by MTT assay. **c** Different lung cancer cell lines were treated with 10 μM DHA-37 for 48 h and then the cell viability was measured by MTT assay. **d** A549 cells were treated with DHA-37 at indicated concentrations for 12, 24, 48, and 72 h, respectively. Cell viability was measured as above. **e**, **f** A549 cells were treated with indicated concentrations of DHA-37 for 24 h and then cells were analyzed by flow cytometry. Gates M1, M2, and M3 indicated G0/G1 phase, S phase, and G2/M phase, respectively. The data are presented as the mean ± SD of three independent experiments. *p < 0.05, **p < 0.01

The cytotoxic effects of DHA-37 and DHA (chemical structure shown in Fig. 1a) on five human cancer cells were examined. Our result showed that DHA-37 could effectively inhibit human lung carcinoma A549 cell, human stomach cancer SGC-7901 cell, human cervical carcinoma Hela cell, human breast cancer MDA-MB-231 and MCF-7 cells at the concentration of 10 μM in 48 h. Meanwhile, DHA-37 exhibited a considerably higher cytotoxic effect than DHA did (Fig. 1b). It is shown that 10 µM DHA-37 treatment had no effect on the proliferation of BEAS-2B cells, but it slightly inhibited the proliferation of Calu-3 and SK-MES-1 cells. The proliferation of A549 was significantly inhibited to 50% compared with the untreated group (Fig. 1c). The anticancer effects of DHA-37 and DHA in human lung carcinoma A549 cell were further compared. DHA-37 could inhibit the cell viability in both time-dependent and dose-dependent manners (Fig. 1d and Figure S1). In order to further illustrate whether DHA-37-induced cell death or inhibited cell proliferation or both, a cell cycle analysis was performed by flow cytometry. As shown in Fig. 1e, f, DHA-37 did not induce any obvious cell cycle arrest at concentrations of 1, 5, or 10 µM. There is only a slight increase (about 5%) in G0/G1 phase at 5 or 10 µM, indicating that DHA-37 did not inhibit cell proliferation. This result indicated that the antineoplastic effect of DHA-37 is mainly through inducing cell death.

### Cell death caused by DHA-37 is dependent on autophagy

**Fig. 2:**
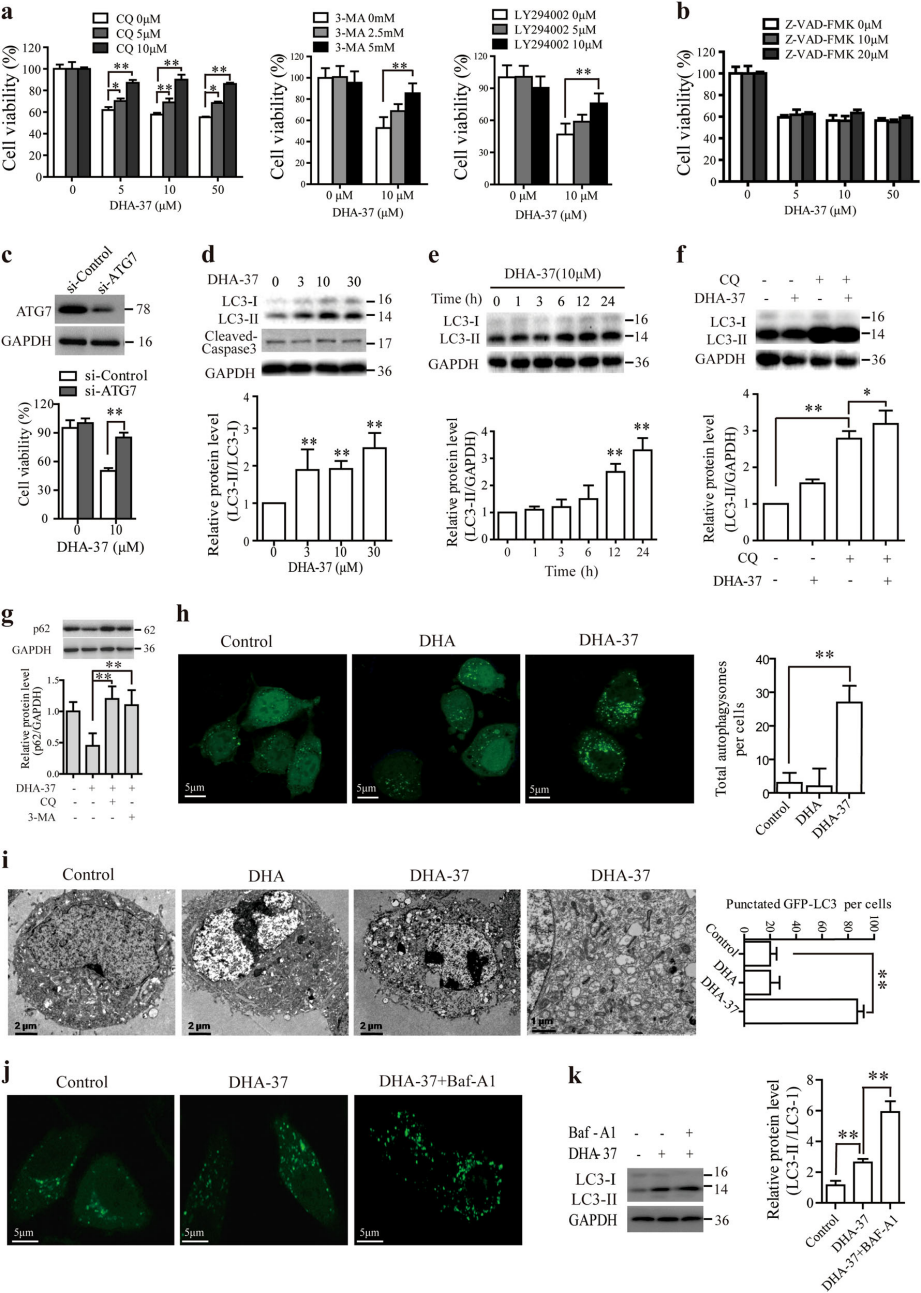
DHA-37 induces the autophagic cell death in non-small-cell lung carcinoma A549 cells. **a**, **b** A549 cells were respectively pre-treated with CQ, 3-MA, LY294002, or Z-VAD-FMK at indicated concentrations for 2 h, then 10 μM DHA-37 was added and co-incubated for 48 h. MTT assay was used to detect the cell viability. **c** A549 cells were transfected with control siRNA or siRNA targeting ATG7. The knockdown of ATG7 was confirmed by western blotting. ATG7 downregulated A549 cells were treated with 10 μM DHA-37 for 48 h, and the cell viability was measured by MTT assay. **d** A549 cells were treated with DHA-37 at indicated concentrations for 24 h before cell lysis. Cell extracts were analyzed by western blotting. **e** A549 cells were treated with 10 μM DHA-37 at indicated time before cell lysis. Cell extracts were analyzed by western blotting. **f** A549 cells were pre-treated with 10 μM CQ for 2 h, then 10 μM DHA-37 was added and coincubated for 24 h. Cell extracts were analyzed by western blotting. **g** A549 cells were cultured with indicated concentrations of DHA-37 for 24 h. Then, the expression levels of p62 were analyzed by western blotting. **h** pcDNA3.1-LC3-GFP and pcDNA3.1 were transfected into A549 cells and then cells were treated with 10 μM DHA-37 and DHA, respectively, for 24 h. The formation of vacuoles containing GFP-LC3 (dots) was examined by confocal microscopy. **i** Cells were treated with 10 μM DHA-37 or DHA, respectively, for 24 h, cells were then fixed and the images were acquired with scanning electron microscope. **j** pcDNA3.1-LC3-GFP transfected A549 cells were treated with DHA37 for 24 h in the presence or absence of bafilomycin A1. The formation of vacuoles containing GFP-LC3 (dots) was examined by confocal microscopy. The endogenous expression levels of LC-3 were analyzed by western blotting. **k** The data are presented as the mean ± SD of three independent experiments. *p < 0.05, **p < 0.01

It is reported that apoptosis is the possible mechanism for DHA-induced cell death. However, our early studies revealed that treatment of the A549 cells with DHA-37 for 24 or 48 h did not notably influence the apoptosis population, indicating that there was an alternative cell death pathway involved in DHA-37-induced cell death. To investigate this problem, three cell death-related pathway (apoptosis, autophagy, and necroptosis) inhibitors were used. The A549 cells were pretreated with apoptosis inhibitor Z-VAD-fmk, autophagy inhibitor CQ, 3-MA or LY294002, necroptosis inhibitor Nec-1, respectively, and subsequently treated with DHA-37, and then the cell death was assessed. Interestingly, the growth inhibitory effect of DHA-37 was blocked significantly by autophagy inhibitors (Fig. 2a), but not apoptosis or necroptosis inhibitors (Fig. 2b and Figure S2, S3), which provides a strong piece of evidence for an autophagy-dependent cell death involved in DHA-37-treated cells.

To further confirm whether DHA-37-induced cell death is dependent on autophagy in A549 cells, the core autophagy regulators ATG7 were knockdown by siRNA transfection. It is shown that DHA-37-induced cell death was significantly inhibited by a knockdown of ATG7 gene expression (Fig. 2c), suggesting that autophagy is a kind of cell death mechanisms. Next, the production of LC3-II was examined, which is used as a reliable marker of autophagy^26^. As shown in Fig. 2d, the amount of LC3-II increased markedly with the rising dose of DHA-37 over 24 h in A549 cells. The increment of LC3-II also displayed a time-dependent manner (Fig. 2e). In agreement with this observation, western blot analysis also showed that pretreatment with CQ resulted in the accumulation of LC3- II with DHA-37 treated or untreated (Fig. 2f). As CQ acts on the late stage of autophagy, the presence of CQ will block the autophagic flux and thus enhance the level of LC3-II.

p62 acts as an adapter protein linking LC3 with ubiquitin moieties. Autophagy therefore mediates the clearance of p62^27^. The p62 level was detected by western blot. As shown in Fig. 2g, A549 cells were treated with DHA-37 for 24 h, the expression levels of p62 were downregulated, and both autophagy inhibitors CQ and 3-MA could reverse DHA-37-induced p62 downregulation.

The formation of LC3 puncta is another marker of autophagosomes. Next, the green fluorescent protein-fused LC3 (GFP-LC3) was used to detect autophagy. As shown in Fig. 2h, treatment with 10 μM DHA-37, but not DHA, could markedly increase the formation of GFP-LC3-labeled vacuoles in A549 cells. Similar results were also observed using transmission electron microscopy that the accumulation of numerous lamellar structures and double-membraned cytosolic autophagic vacuoles in A549 cells were detected after treatment with DHA-37, but not DHA (Fig. 2i). To further investigate whether DHA-37-induced autophagy initiation or blocked autophagosome degradation, A549 cells were treated with DHA37 for 24 h in the presence or absence of bafilomycin A1. As shown in Fig. 2j, k, bafilomycin A1 treatment led to an increase in the amount of both LC3-II and GFP-LC3 dots. Collectively, these findings prove that distinct from DHA-induced apoptosis, the cell death caused by DHA-37 is dependent on autophagy. Together with the results that DHA-37-induced cell death was remarkably inhibited by 3-MA, these results indicate that DHA-37 induced the enhancement of autophagic initiation.

### HMGB1 involved in DHA-17-induced autophagic cell death

**Fig. 3:**
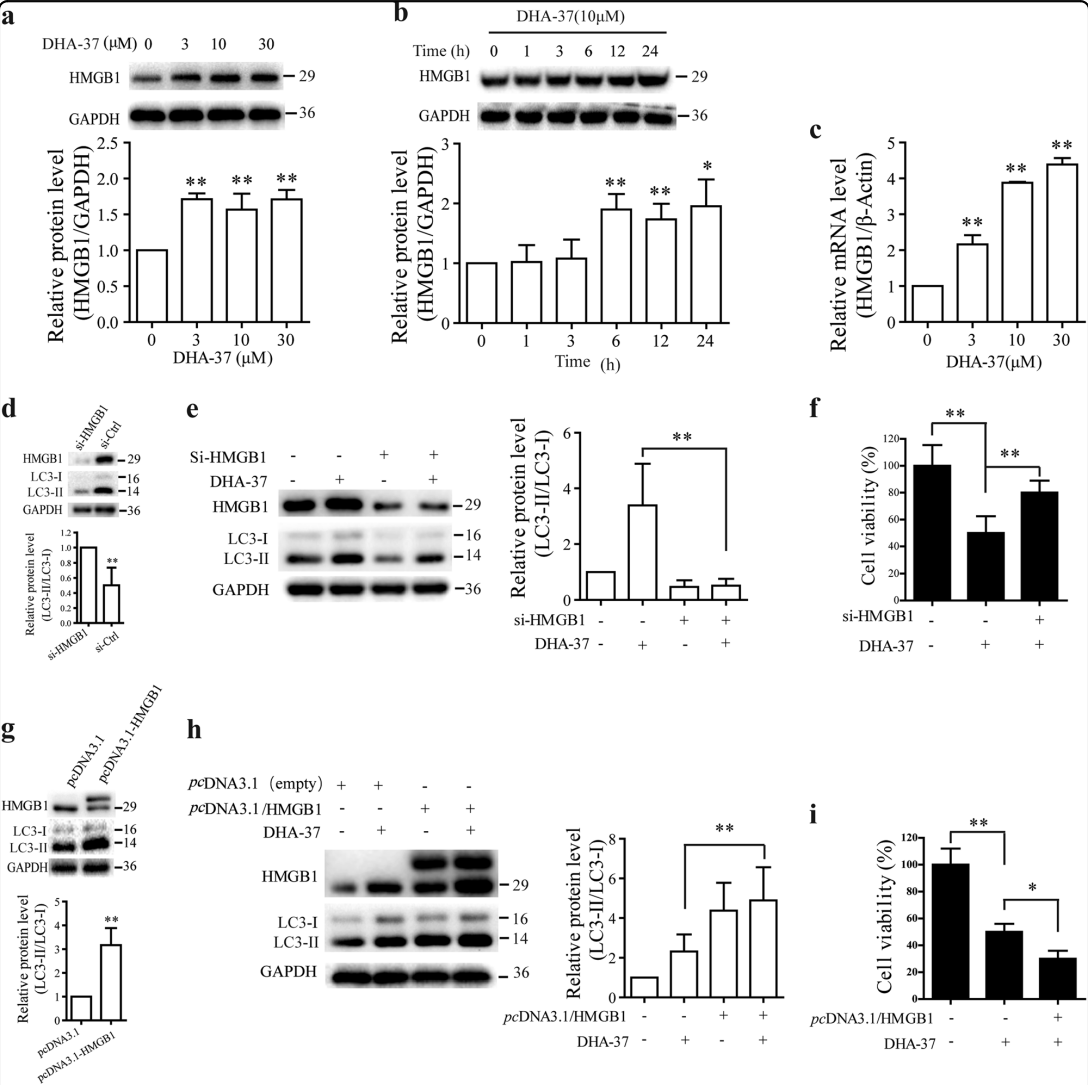
HMGB1 involved in DHA-37-induced autophagic cell death in A549 cells. **a** A549 cells were treated with DHA-37 at indicated concentrations for 24 h before cell lysis. Cell extracts were analyzed by western blotting. **b** A549 cells were treated with 10 μM HA-37 at indicated time before cell lysis. Cell extracts were analyzed by western blotting. **c** A549 cells were treated with DHA-37 at indicated concentrations for 24 h and total RNA was extracted, mRNA levels were detected using quantitative polymerase chain reaction (Q-PCR). **d**, **e** A549 cells were transfected with control siRNA or siRNA target HMGB1. The knockdown of HMGB1 was confrmed by western blot (**d**). The cells were treated or not with 10 μM DHA-37 for 24 h. Cell extracts were analyzed by western blotting (**e**). **f** A549 cells were transfected or not with siRNA targeting HMGB1 and control siRNA, respectively. After 24 h, cells were treated with 10 μM DHA-37 for 48 h, and cell viability was measured by MTT assay. **g**, **h** A549 cells were transfected with *pc*DNA3.1-HMGB1 or *pc*DNA3.1 for 24 h, respectively, Cells were treated or not with 10 μM DHA-37 for 24 h, the cell extracts were analyzed by western blotting. **i** A549 cells were transfected or not with HMGB1 over-expression vector and empty vector, respectively. After 24 h, cells were treated with 10 μM DHA-37 for 48 h, and cell viability was measured by MTT assay. The data are presented as the mean ± SD or SEM. *n* ≧ 3. *p < 0.05, **p < 0.01

Growing evidence suggests that artemisinin and its derivatives exert antitumor action associated with enhanced levels of oxidative stress. Since oxidative stress is a central regulator of HMGB1’s activity in cell death and HMGB1 is an autophagy sensor^17,28,29^, we wondered whether DHA-37-induced autophagic cell death is due to the modulation of HMGB1. So, the protein levels of HMGB1 were examined first. As shown in Fig. 3a, HMGB1 protein expression level increased markedly with the rising dose of DHA-37 over 24 h in A549 cells. The HMGB1 increase also displayed a time-dependent manner (Fig. 3b). To further investigate whether DHA-37 regulates HMGB1 at transcriptional level, the HMGB1 mRNA levels were detected by Q-PCR assay. As results shown in Fig. 3c, increasing concentrations of DHA-37 (1, 3, 10, and 30 µM) can promote the transcription of HMGB1 in A549 cells effectively.

Next, we examined whether silencing or overexpression of *HMGB1* genes could affect DHA-37-induced autophagy or autophagic cell death in A549 cells. RNAi experiment showed that silencing HMGB1 by siRNA knockdown (Fig. 3d) decreased the LC3-II/LC3-I ratio significantly and reversed the increase of LC3-II/LC3-I ratio induced by DHA-37 (Fig. 3e). MTT assay also showed that downregulation of HMGB1 expression could reverse DHA-37-induced cell death significantly (Fig. 3f). Conversely, overexpression of HMGB1 (Fig. 3g) by transient transfection in A549 cells enhanced the DHA-37-induced increase of LC3-II/LC3-I ratio significantly (Fig. 3h) and cell death (Fig. 3i).

Taken together, these results suggest that HMGB1 involved in DHA-37-induced autophagic cell death in A549 cells.

Since DHA-37 can increase the transcription level of HMGB1 in a dose-dependent manner (Fig. 3c), we wonder if DHA-37 regulates the transcription of HMGB1 through modulating one of the transcription factors. Therefore, the A549 cells were treated with DHA-37, and then all the reported transcription factors which could regulate the transcription of HMGB1 were detected by a Q-PCR assay (data not shown). Among them, the KLF4 is the most highly upregulated transcription factor by DHA-37 treatment. Q-PCR and western blot analysis showed that DHA-37-induced KLF4 could increase both in protein and mRNA levels (Figure S4a and b). These data indicate that KLF4 may contribute to DHA-37-induced HMGB1 upregulation.

### DHA-37 induces autophagic cell death through MAPK-dependent and PI3K-Akt1–mTOR-independent signaling pathways

**Fig. 4:**
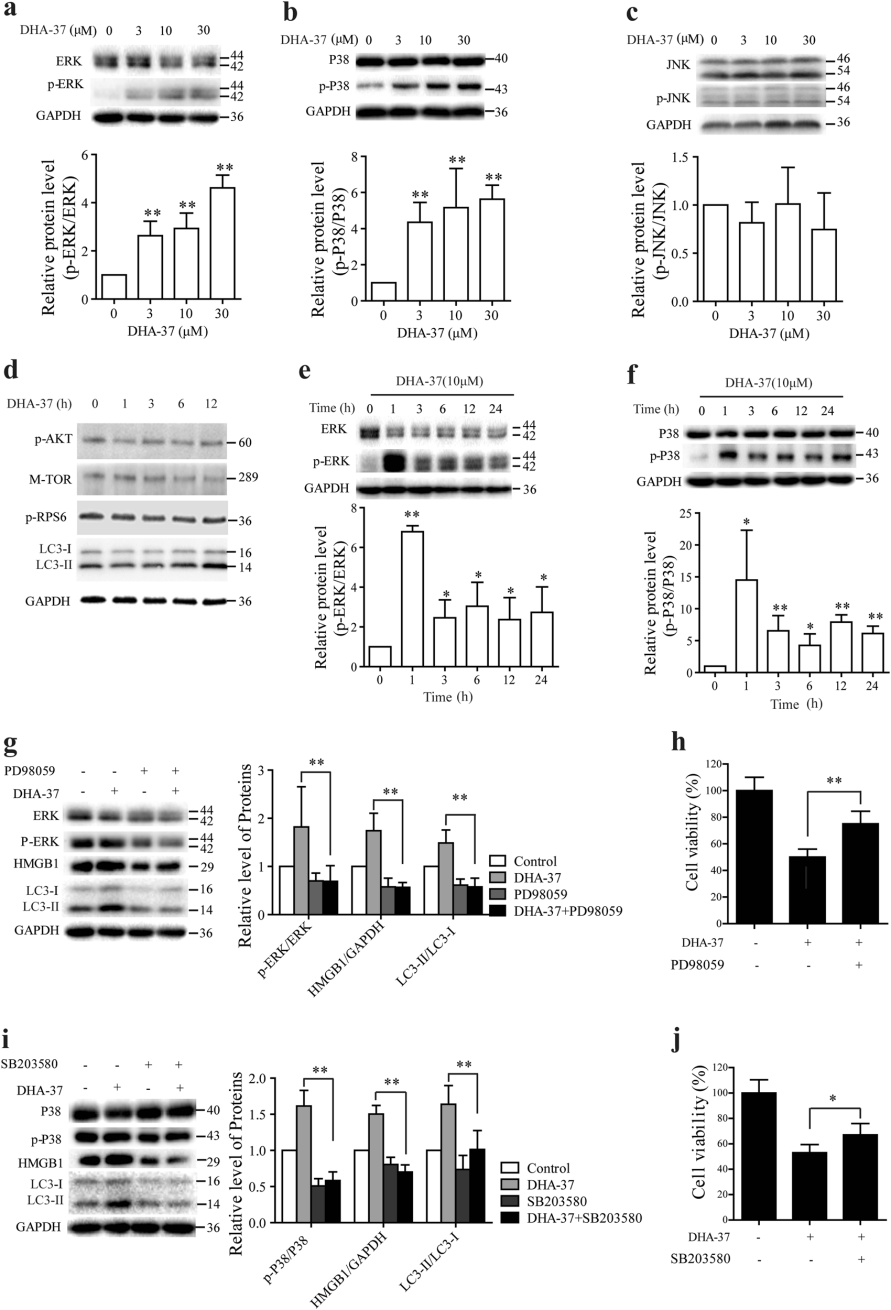
DHA-37 induces autophagic cell death through ERK1/2 and p38-dependent signaling pathways. **a**, **b**, **c** A549 cells were treated with DHA-37 at indicated concentrations for 24 h. Cell extracts were analyzed by western blot for phosphorylated and total ERK (**a**), p38 (**b**), and JNK expression (**c**). **d** A549 cells treated with 10 μM DHA-37 for various time intervals were analyzed by western blot. The results shown are representative of three experiments. **e**, **f** A549 cells with or without 10 μM DHA-37 treatment for indicated times were analyzed by western blot for phosphorylated and total ERK (**e**) and p38 expression (**f**). **g** A549 cells pretreated with 10 μM PD98059 and/or DHA-37 for 24 h were analyzed by western blot using indicated antibodies. **h** A549 cells were treated with PD98059 and/or 10 μM DHA-37 for 48 h. Cell viability was measured by MTT assay. **i** A549 cells were pretreated with 20 μM SB203580 or not for 1 h, then 10 μM MDHA-37 was added into cell culture for 24 h. Cell extracts were analyzed by western blotting. **j** A549 cells were treated with SB203580 and/or 10 μM DHA-37for 48 h. Cell viability was measured by MTT assay. The data are presented as the mean ± SD. *n* ≧ 3. *p < 0.05, **p < 0.01

There are two major pathways involved in the regulation of autophagy including the PI3K/Akt1/mTOR pathway and the MAPK pathway^30–32^. To further clarify the mechanism of DHA-37-induced autophagy, the effects of DHA-37 on both pathways using western blotting and MTT assay were examined. A549 cells were treated with various concentrations of DHA-37 for 24 h, and there was an increase in the phosphorylation of MAPK1 (ERK2)/MAPK3 (ERK1) (Fig. 4a), and p38 (Fig. 4b). However, MAPK8 (JNK1) was not activated by the same treatment of DHA-37 (Fig. 4c). Subsequently, whether DHA-37 also activates the PI3K/Akt1/mTOR pathway was examined. As shown in Fig. 4d, there was no obvious change in the phosphorylation of AKT1 (Thr308), mTOR (Ser2448), and its downstream targets RPS6 (ribosomal protein S6). These preliminary data suggest that MAPK1 (ERK2)/MAPK3 (ERK1) and p38 pathway, but not PI3K-Akt1–mTOR, were involved in the cytotoxic effect of DHA-37.

To further confirm the role of MAPK1 (ERK2)/MAPK3 (ERK1) and p38 in DHA-37-induced autophagic cell death, A549 cells were treated with 10 μM DHA-37 and the time course of ERK 1/2 and p38 phosphorylation were detected. As shown in Fig. 4e, f, treatment with DHA-37 led to a strong phosphorylation of ERK1/2 and p38 within 1 h and persisted up to 24 h. Subsequently, phosphorylation inhibitors of ERK1/2 and p38 were employed. As expected, ERK1/2 inhibitor PD98059 reversed the increasing LC3-II/LC3-I ratio (Fig. 4g) and prevented DHA-37-induced increase of HMGB1. As expected, ERK1/2 inhibitor PD98059 prevented DHA-37-induced cell death (Fig. 4h).

Similar results were observed after inhibiting the phosphorylation of p38 with SB203580 (Fig. 4i, g). These data demonstrate that ERK1/2 and p-P38 function as upstream of autophagy involved in the DHA-37 could induce the increase of HMGB1 and autophagic cell death in A549 cells.

### DHA-37 shows antitumor efficacy in A549 xenograft lung cancer mode

**Fig. 5:**
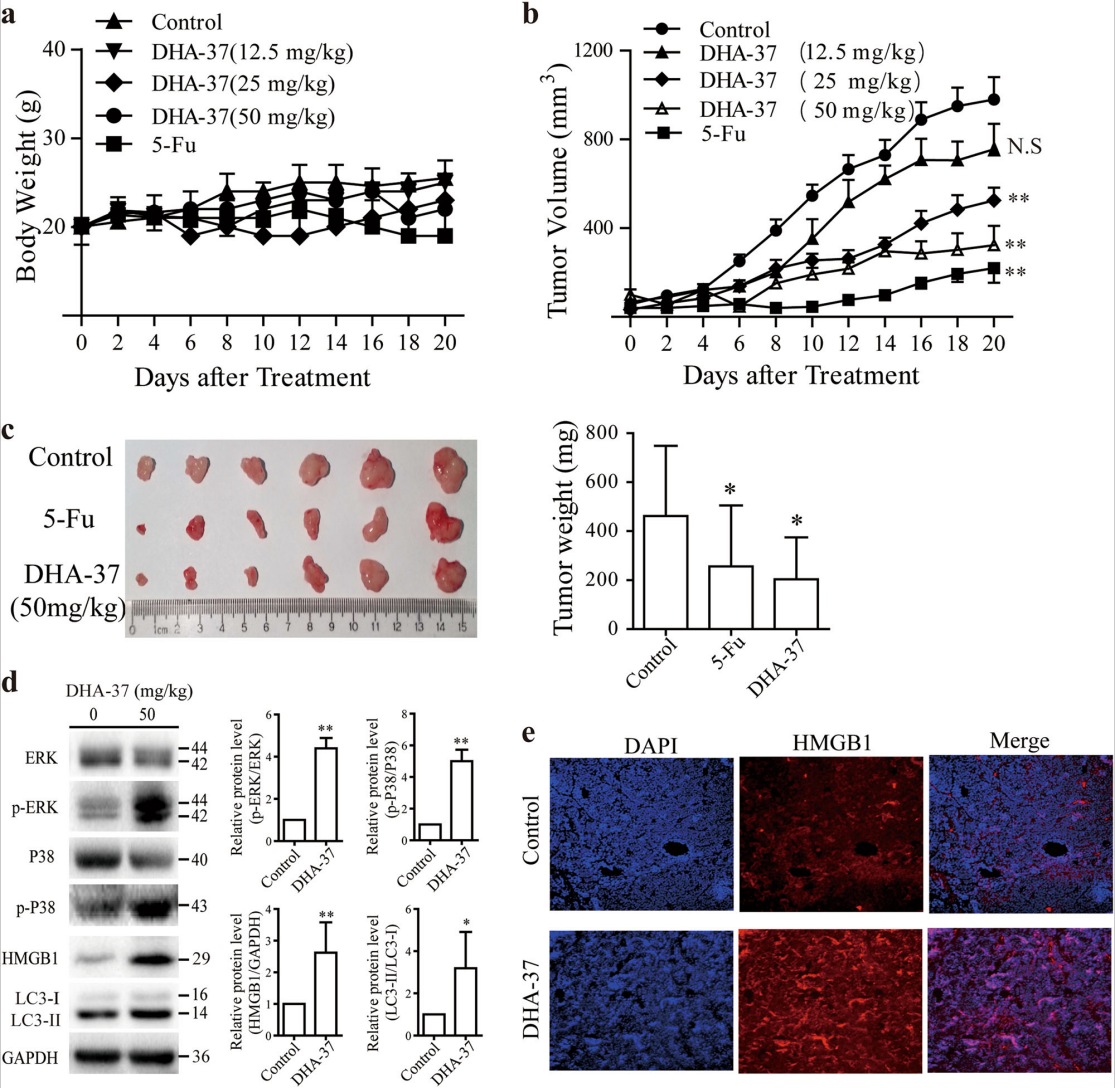
Effects of DHA-37 on A549 tumor xenografts. Nude mice bearing an A549-derived tumor were treated with DHA-37 or 5-Fluorouracil (5-fu). **a** Body weights of mice were measured. **b** Tumor volumes were measured every day. **c** Mice were killed and tumors were then photographed and weighed. **d** Tumor lysates for western blot analyses by using indicated antibodies. **e** Immunofluorescence staining was performed to investigate HMGB1 expression in tumor tissues. The data are presented as the mean ± S.D. *n* = 8. *p < 0.05, **p < 0.01

Lastly, the above in vitro findings were validated in vivo in a human A549 lung cancer xenograft model. A549 cells were subcutaneously injected into the flank of nude mice, and then treated with DHA-37 or 5-FU (positive control), until small palpable tumors arrived to 50–100 mm^3^. The tumor diameters and body weight were measured, and then the tumors were harvested for western blot analysis of proteins of interest when tumors reached 1000 mm^3^. As shown in Fig. 5a, no obvious body weight loss was observed by treatment with 50 mg/kg DHA-37, while tumor growth was inhibited significantly (Fig. 5b), and the maximum tumor weight inhibitory rate reached to 56.01% (Fig. 5c). After treatment for 20 days, mice were killed immediately for the humanitarian reasons. The expression of p-ERK, p-P38, HMGB1, and LC3 in tumors were detected by western blot. As shown in Fig. 5d, the protein level of p-ERK, p-P38, HMGB1, and LC3 increased significantly comparing with control group. As expected, DHA-37 induced a significant increase of HMGB1 expression in tumor tissue (Fig. 5e). Collectively, daily intraperitoneal administration of DHA-37 at 25/50 mg/kg for 20 days showed a good tumor inhibition effect. Meanwhile, the cell signal points activated in cell level are also activated in vivo.

## Discussion

In this study, we provide evidences that a novel DHA derivative DHA-37 exhibits more excellent anticancer activity than DHA in several cancer cell lines. Moreover, based on the observation that autophagy inhibitor CQ, 3-MA, or bafilomycin A1 could reverse DHA-37-induced cell death, and the apoptosis inhibitor Z-VAD-fmk and necroptosis inhibitor Nec-1 have no effect on DHA-37-induced cell death in A549 cells. We proposed that DHA-37 activated the autophagic cell death, which is different from apoptosis induced by DHA. The proposed conclusions have been further validated by analyzing the punctate distribution of eGFP-LC3 and the ratio of LC3-II/LC3-I in DHA-37-treated A549 cells. We also demonstrate that HMGB1 is essential for DHA-37-induced autophagy. Both the mRNA and protein level of HMGB1 increased significantly by DHA-37 treatment. Further, we show that knockdown of HMGB1 by siRNA transfection could reverse DHA-37-induced cell death. We also demonstrate that the activation of MAPK1 (ERK2)/MAPK3 (ERK1) and p38 were involved in the cytotoxic effect of DHA-37 (Fig. 4). Finally, the in vitro results were validated in an animal model. DHA-37 showed anticancer activity in A549 xenograft mice model. The activation of autophagy marker LC3 and the increased expression of HMGB1 were also observed in the animal study. Moreover, the MAPK signaling pathways involved in DHA-37-induced autophagy in cell culture were indeed activated in the tumors of DHA37-treated mice. These results indicated that the findings of the in vivo study were well consistent with those results in the in vitro study.

ART and its derivatives (such as DHA, artesunate, and artemether) are attractive anticancer drugs due to their selective toxicity toward cancers and clinical safety. However, their anticancer activity is relatively low (IC_50_ at the micromolar range) compared with its anti-malaria parasites activity (IC_50_ up to 15 nM)^33,34^. Therefore, the design and synthetic of novel derivatives with enhanced antitumor activity are needed. In our previous studies, a series of dihydroartemisinin-cinnamic acid ester derivatives modified on C-12 and/or C-9 position(s) were synthesized^7,8^. Among them, DHA-37 exhibited a better anticancer activity than DHA or other derivatives. In this study, we further compared the anticancer effect between DHA and DHA-37 in different cancer cell lines. Consistent with the pervious study, DHA-37 exhibits better antitumor activity than DHA (Fig. 1b). In A549 lung cancer cells, DHA exhibits IC_50_ at 80.42 μM, while DHA-37 decreased the IC_50_ to 0.20 μM and was close to the lowest levels of monomeric artemisinin derivatives arrived. It has been reported that a series of DHA monomeric derivatives via an aza-Michael addition reaction with high selectivity index and with a IC_50_ at 0.37 μM in HeLa cells^35^. It has been reported that DHA could induce apoptosis via modulating the cytochrome *c* release, Bax overexpression, increase in Bax/Bcl2 ratio, and activation of caspases 3, 8, and 9^34,36-38^, and also there is no doubt that the induction of apoptosis is an effective strategy for cancer control. However, it is interesting that there was no dramatic increase in the number of apoptosis cells after DHA-37 treatment of A549 human lung cancer cells (about 10% apoptosis rate)^7^. It means other non-apoptosis death pathways were involved in DHA-37-induced cell death. In this study zVAD-fmk was used to block the apoptosis pathway, and it is shown that apoptosis is not the main form for DHA-37-induced cell death. Nec-1 was also been used to exclude the possibility of DHA-37-induced necroptosis. Autophagy inhibitors were used to confirm that DHA-37 could induce autophagic cell death. However, in the past decade, there are also some other forms of cell death mechanisms are described such as oncosis, pyroptosis, entosis, mitotic catastrophe and so on. We cannot exclude if other zVAD- or Nec-1-insensitive cell death pathways are involved in DHA-37-induced cell death.

On the one hand, the inhibition of autophagy could increase the sensitivity of cancer cell to apoptosis-inducing agents^39,40^. On the other hand, excess autophagy could induce the autophagic cell death. It has been reported that several chemotherapeutic agents could induce autophagic cell death^14–16^. Consistent with these compounds, we found that DHA-37 activated the autophagic cell death (Fig. 2), which is different from apoptosis induced by DHA^7^. This is a new promise of DHA-37 acting as an effective and safe anticancer drug to treat apoptosis-resistant cancer. Overall, although the controversy still exists and the “double face” role of autophagy in cancer therapy, autophagy is becoming an attractive target for anticancer therapies.

HMGB1 was first isolated as a DNA-binding protein, locating in the nuclear. Its main function is to regulate the gene expression. Subsequent studies showed that HMGB1 could translocate to the cytoplasm or release to the outside of the cell, and act as a stress sensor involved in various physiological and pathological processes, such as inflammation, immunity, and cancer^41–43^. Our results showed that DHA-37-induced autophagy via an HMGB1-dependent manner. DHA-37 could increase dramatically the HMGB1 expression in both time- and dose-dependent manners. Downregulation of HMGB1 by siRNA could reverse DHA-37-induced autophagy (Fig. 3). This is consistent with other groups reported that HMGB1 is required for autophagy^20,22^. However, there is still some controversy over whether the autophagy is HMGB1 dependent or independent. For example, Schwabe et al.^23^ reported that HMGB1 is dispensable for autophagy. Based on the observation that conditional liver knockout of HMGB1 in GFP-LC3 mice has no effect on GFP-LC3 puncta and GFP-LC3 cleavage. In contrast, in other studies, knockout of HMGB1 in myeloid cells showed a protective function against endotoxemia and bacterial infection, which is through downregulation of autophagy^42^. We speculate that the role of HMGB1 in autophagy may be cell or organ dependent. Development of anticancer drugs targeting HMGB1-induced autophagy needs to further determine the relationship between HMGB1 and autophagy against different cancer types. Overall, although the role of HMGB1 in cancer therapy is complex, HMGB1 is becoming an attractive target for anticancer therapy.

**Fig. 6:**
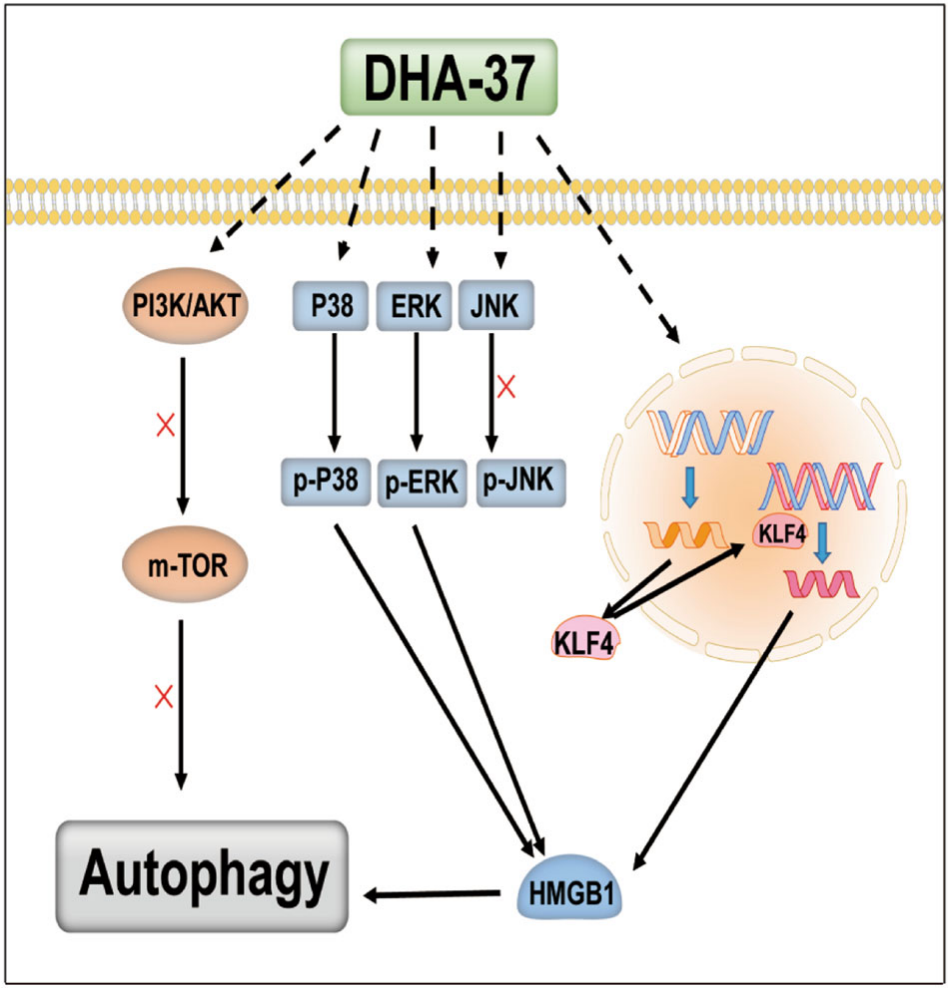
Schematic of the proposed mechanism of DHA-37-inducing autophagic cell death in A549 cells via the MAPK pathway and an HMGB1-depedent manner. (See text for details)

It was reported that PI3K/mTOR/Akt and MAPK pathways are two of the major pathways involved in the regulation of autophagy^44^. In the present study, we found that both the phosphorylation of AKT, mTOR, and downstream protein PRS6 cannot be inhibited in the DHA-37-treated cells (Fig. 4d). These findings indicate that PI3K/mTOR/Akt pathway is not involved in DHA-37-induced autophagic cell death. We observed that 1 h after DHA-37 administration, the phosphorylation of MAPK1 (ERK2)/MAPK3 (ERK1) (Fig. 3c), and p38 (Fig. 3d) increased dramatically. We also observed that inhibiting the activation of p38 and ERK1/2 could reverse the increased expression of HMGB1 and LC3-II to LC3-I ratio. Based on the present results, we propose that DHA-37 could induce autophagic cell death through the activation of MAPK. The p38 and ERK1/2 activation is an essential upstream signal to trigger autophagy. As shown in Fig. 6, graphic model for DHA-37-induced autophagic cell death is represented.

Overall, our data suggest that DHA-37-induced cell death in 549 human non-small-cell lung carcinoma cells is mediated by the autophagic cell death. The chemical structure of DHA-37 could serve as a “template” for the development of novel artemisinin derivatives as effective chemotherapeutic agents against the apoptosis-resistant cancers.

## Materials and methods

### Antibodies and reagents

The antibodies against LC3B (2775), HMGB1 (6893), phospho-mTOR (Ser2448) (2971), phospho-AKT (Thr308) (4056), phospho-RPS6/S6 ribosomal protein (Ser235/236) (2211), phospho-MTOR (2971), MAPK1 (ERK2)/MAPK3 (ERK1) (9102), phospho-MAPK1/3 antibody (Thy202/Tyr204) (9101), p38 (9212), phospho-p38 (Thr180/Tyr182) (4631), MAPK8 (JNK1) (9252), phospho-MAPK8 (Thr183/Tyr185) (4668), and ATG7 (2631) were purchased from Cell Signaling Technology. Anti-GAPDH antibody, anti-SQSTM1/p62 (P0067) antibody, chloroquine (CQ), and Nec-1 were purchased from Sigma-Aldrich. Anti-HMGB1 antibody (EPR3507) was purchased from Abcam. Goat polyclonal KLF4 antibody was purchased from BD. 3-MA, Baf-A1, Z-VAD-FMK, and LY294002 were the product of selleck (Shanghai, China), PD98059 and SB203580 were purchased from Calbiochem (CA, USA). DHA was purchased from DEMO Medical Tech (Shanghai, China). 5-Fu was purchased from Sunshine Biotechnology (Jiangsu, China). Secondary antibodies for western blot were from Cell Signaling Technology. Secondary antibodies for immunofluorescence were from Jackson ImmunoResearch Laboratories (PA, USA).

### Cell culture

The human lung cancer cell line A549, H1650, Calu-3, SK-MES-1, the human cervical cancer cell line HeLa, the human breast cancer cell line MDA-MB-231, the human breast cancer cell line MCF-7, and the human gastric cancer cell line SGC-7901 cells were cultured with standard methods in DMEM containing 10% FBS (GIBCO, CA, USA) fetal bovine serum and supplemented with 100 U/ml penicillin and 100 U/ml streptomycin.

### Gene transfection and RNAi

A549 cells were split 1 day before transfection to 50% confluence. Si-ATG7, Si-HMGB1, and *pc*DNA3.1/HMGB1 were transfected into A549 cells using ExFect Transfection Reagent of Vazyme (Jiangsu, China) according to the manufacturer's instructions. The final concentration of siRNAs was 300 pM. The final concentration of *pc*DNA3.1/HMGB1 and *pc*DNA3.1-GFP-LC3 were 5 μg per well of six-well plate. HMGB1 siRNA were as follows: 5′-AGA CCU GAG AAU GUA UCC CCA AAdTdT-3′ (sense strand), 5′-UUU GGG GAU ACA UUC UCA GGU CUdTdT-3′ (antisense strand). The nonspecific RNA duplexes and *pc*DNA3.1 were used in control experiments. Cells were harvested after incubation for 48 h, and then western blotting was performed to detect transfection effect.

### Cell viability assay

Cell survival was determined by MTT assay. MTT stock solution (BIOSHARP, Anhui, China) medium at the final concentration of 0.5 mg/ml and incubated at 37 °C for 2–4 h. Medium was replaced with 150 μl DMSO and placed on a plate shaker for 10 min. Absorbance was read at 560 nm and a reference measurement at 650 nm. Readings were performed with the Epoch microplate reader (VT, USA).

### Cell cycle analysis

A549 cells treated with DHA-37 for 24 h. After incubation, cells were harvested, washed with cold PBS, and fixed with 70% ethanol for 24 h. Then, the cells were washed with PBS, stained with a propidium iodide (PI) staining solution containing 100 μg/ml PI, 100 μg/ml RNase A, and 0.1% glucose for 40 min, and then subjected to flow cytometric analysis.

### Assessment of autophagy

pcDNA3.1-LC3-GFP were transfected into A549 cells according to method of gene transfection. A549 cells were treated with DHA-37 at the indicated concentrations after transfection. After 24 h, cells were fixed in 10% PFA for 10 min. Nucleus were stained with DAPI purchased from Beyotime (Jiangsu, China). LSM700 (Carl Zeiss) was used to observe cytomorphology and score green vesicles. The number of vesicles per cell in GFP-positive cells was determined. Approximately 100 cells per sample were counted for triplicate samples, all coverslips were scored with the observer blinded to the identity of the slides.

### Quantitative PCR

Total RNA from A549 cells was extracted using Trizol (Invitrogen, CA, USA). The cDNA was synthesized using an oligodT primer (TransGen, Beijing, China) in a total volume of 20 μl according to the manufacturer's instructions. PCR was performed using an ABI 7900 Sequence Detection System and SYBR Premix EX Taq (TransGen). The primer pairs used were as follows: HMGB1: 5′-GATGGGAAAGGAGATCCTA-3′ and 5′-CTTGGTCTCCCTTTGGGG-3′. β-actin: 5′-AAGAGAGGCATCCTCACCCT-3′ and 5′-TACATGGCTGGGGTGTTGAA-3′. KLF4: 5′-CGAACCCACACAGGTGAGAA3′, and 5′-GAGCGGGCGAATTTCCAT-3′. The mRNA levels were normalized to β-actin. The foldchange for each gene was calculated by comparing the cyclethreshold^26^ value of the gene with the Ct value of the control.

### Western blotting

Cells were collected and suspended in cold RIPA buffer. For tumor tissues, tissues were excised, minced, and then they were homogenized in RIPA buffer. The protein concentration was measured using a BCA protein assay kit (Pierce, 23227). Equal amounts of protein were separated by SDS-PAGE and transferred to polyvinylidene fluoride membranes (Roche Applied Science). The membranes were blocked with 5% BSA and incubated with the primary antibodies, and subsequently with horseradish peroxidase-conjugated secondary antibody. GAPDH levels were analyzed as controls for protein loading. Band intensity was quantifed by BandScan software (BioRAD).

### Transmission electron microscope assay

Cells were harvested in a 1.5 ml microcentrifuge tube for each sample. Cells were washed with cold PBS and fixed for 1 h at 4 °C in 1.6% glutaraldehyde in PBS (pH 7.2). The cells were then washed and fixed again in aqueous 2% osmium tetroxide, dehydrated in ethanol, embedded in Epon, and processed for electron microscopy with a Zeiss EM 902 transmission electron microscope at 80 kV. Ultrathin sections were cut and stained with uranyl acetate and lead citrate.

### Animal experiments

All surgical procedures and care administered to the animals were in accordance with institutional guidelines. Male nude BALB/c mice, 4–6 weeks old, were obtained from the Experimental Animal Center at Yangzhou University. A total of 2 × 10^6^ of A549 cells were suspended in sterile PBS and injected subcutaneously into the right flank of the mice. When tumors reached around 50–100 mm^3^, the hosting mice were randomly divided into five groups (8 in each group): (a) control (DMSO was dissolved in 200 μl corn oil, once daily by i.p. injection); (b) DHA-37 (12.5 mg/kg, once daily by i.p. injection); (c) DHA-37 (25 mg/kg, once daily by i.p. injection); (d) DHA-37 (50 mg/kg, once daily by i.p. injection); (e) 5-Fu (20 mg/kg, once daily by i.p. injection). The mice were closely monitored and body weights were measured every day. Tumor size was measured every day with calipers and tumor volumes were estimated according to the formula: TV (mm^3^) = AB^2^/2, where A is long axis, and B is the short axis. The mice were closely monitored for 20 days and then killed. Then, tumors were removed, each tumor was split into three pieces: one fixed in 10% PFA, one was used to extract total protein, and the other stored at 80 °C for further analysis.

### Immunofluorescence assay

The expression of HMGB1 in tumor tissues was examined by Immunofluorescence^26^. the tumor tissues were fixed in 4% paraformaldehyde (PFA), embedded in paraffin, and cut into 5 μm thick sections. IF analyses were performed with primary antibodies for HMGB1. Cell nuclei were stained with DAPI. Digital images of IF staining were captured with LSM700 (Carl Zeiss) microscope.

### Statistical analysis

All data were shown as mean ± SD at least three independent experiments except Q-PCR data. Q-PCR data were presented as mean ± SEM from at least three independent experiments. Difference was determined for statistical significance using one-way ANOVA or Student’s *t* test and *p* < 0.05 was considered as statistically significant.

## Electronic supplementary material

SUPPLEMENTAL MATERIAL
