# Supplementary material for: Novel dihydroartemisinin derivative DHA-37 induces autophagic cell death through upregulation of HMGB1 in A549 cells
Source: Cell Death Dis. 2018 Oct 15;9(11):1048. doi: 10.1038/s41419-018-1006-y (PMC6189137; doi:10.1038/s41419-018-1006-y)
Supplement: Supplementary file 1 — SUPPLEMENTAL MATERIAL [file 41419_2018_1006_MOESM1_ESM.docx]

**Novel dihydroartemisinin** **derivative DHA-37 induces autophagic cell death through up-regulation of HMGB1 in A549 cells**

Xiufeng Liu^1,2,3^, Juanjuan Wu^1,3^, Menglin Fan^1^, Chen Shen^1^, Wenling Dai^1^, Yini Bao^1^, Ji-Hua Liu^1,2^* and Bo-Yang Yu^1,2^

^1^ State Key Laboratory of Natural Medicines, School of Traditional Chinese Pharmacy, China Pharmaceutical University, Nanjing 210009, PR China; ^2^ Jiangsu Key Laboratory of TCM Evaluation and Translational Research，China Pharmaceutical University, Nanjing 210009, PR China; ^3^These authors contributed equally to this work.

*Correspondence author: J-H Liu, State Key Laboratory of Natural Medicines, School of Traditional Chinese Pharmacy, China Pharmaceutical University, Nanjing 210009, PR China. Tel/Fax: +86 25 8618 5157; E-mail: liujihua@cpu.edu.cn

KEY WORD: Autophagic cell death, Artemisinin, DHA-37, HMGB1, MAPK

SUPPLEMENTARY FIGURE:


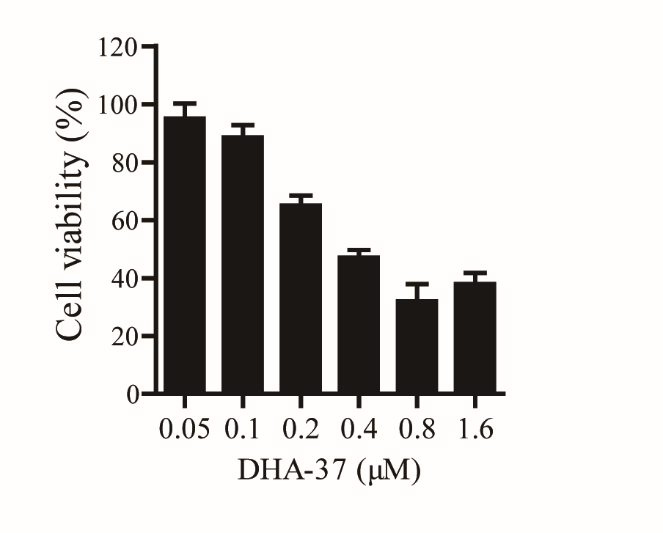


**Figure S1.** A549 cells were treated with DHA-37 at indicated concentrations for 72 h and then the cell viability was measured by MTT assay.


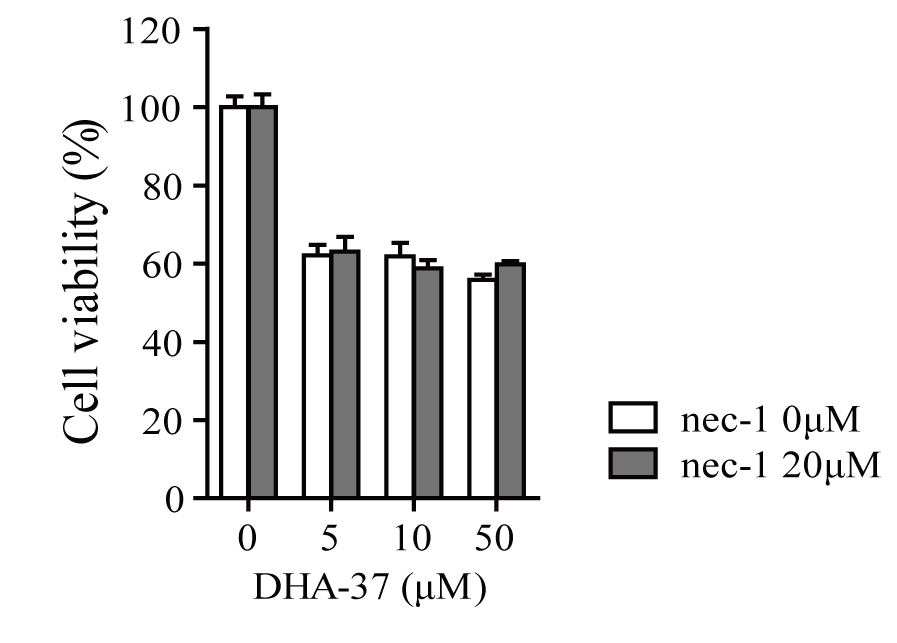


**Figure S2.** A549 cells were pre-treated with nec-1 at indicated concentrations for 2 h, then 10 μM DHA-37 was added and co-incubated for 48 h. MTT assay was used to detect the cell viability.


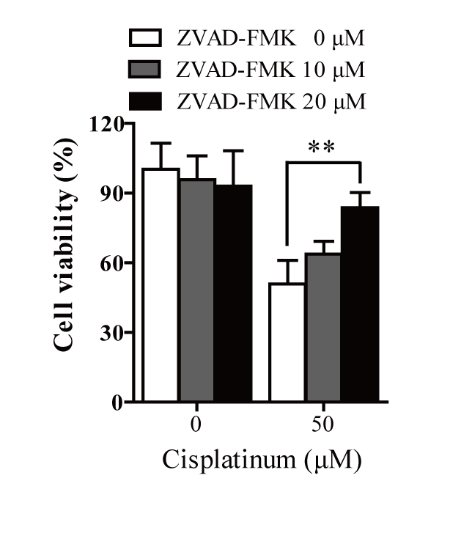


**Figure S3.** A549 cells were pre-treated with Z-VAD-FMK at indicated concentrations for 2 h, then 50 μM cisplatin was added or not and co-incubated for 48 h. MTT assay was used to detect the cell viability.


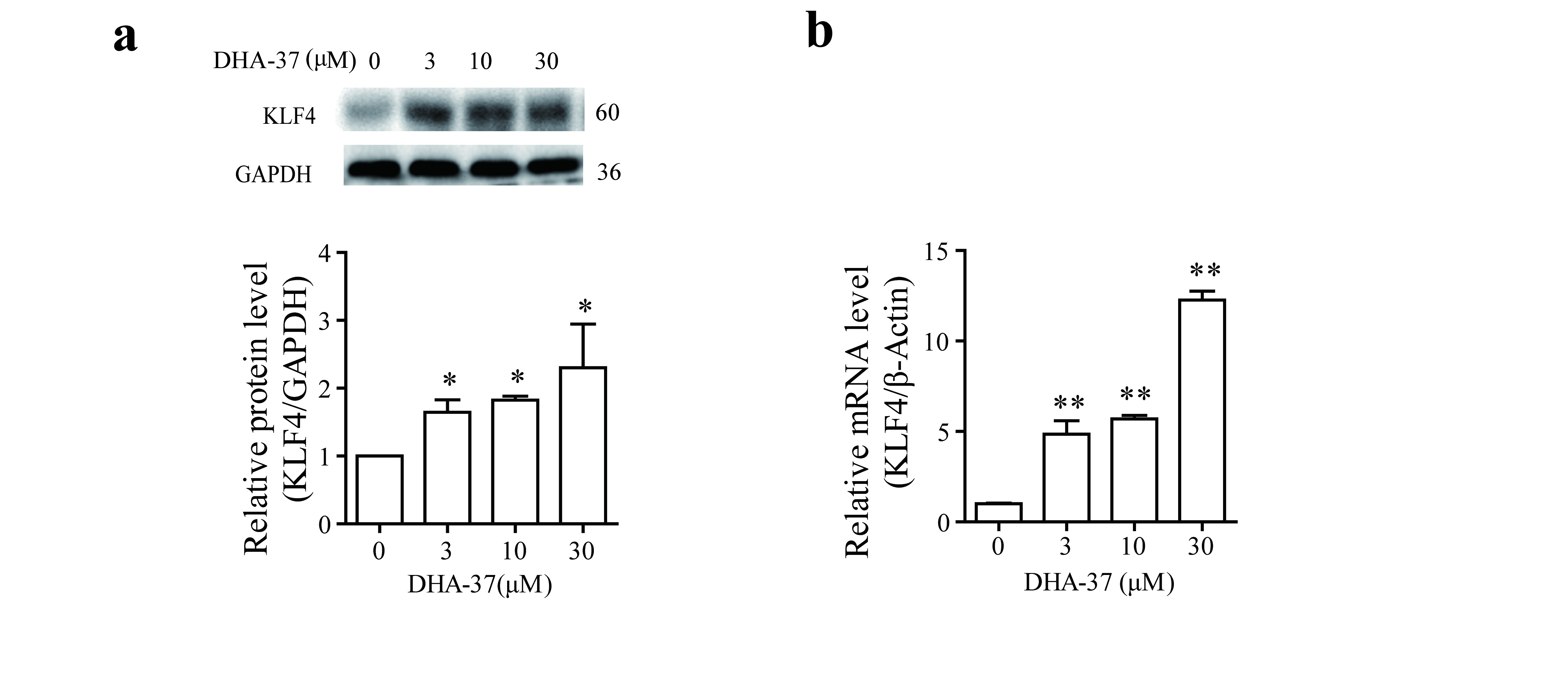


**Figure S4. DHA-37 induced upregulation of *KLF4* expression at both mRNA and protein levels.** A549 cells treated with 10 μM DHA-37 for various time-intervals were analyzed by western blot **(a)** and quantitative polymerase chain reaction (Q-PCR) **(b)** assay. The data are presented as the mean ± SD. n≧3. * *p* < 0.05, ** *p* < 0.01.
